# Comparison of needle and conventional arthroscopy for visualisation of predefined anatomical structures of the knee joint: a feasibility study in human cadavers and patients

**DOI:** 10.1186/s12891-024-07346-9

**Published:** 2024-03-12

**Authors:** Notker Blankenburg, Ralf Henkelmann, Jan Theopold, Sabine Löffler, Pierre Hepp

**Affiliations:** 1 Department of Orthopedic, Trauma and Plastic Surgery, University of Leipzig, Liebigstraße 20, Leipzig, 04103 Germany; 2 Institute of Anatomy, University of Leipzig, Liebigstraße 13, Leipzig, 04103 Germany

**Keywords:** Needle arthroscopy, Knee joint, Arthroscope, Diagnostic arthroscopy, Chip-on-tip image sensor technology, Feasibility study

## Abstract

**Background:**

In terms of the optics used for Knee arthroscopy, a large number of different endoscopes are currently available. However, the use of the 30° optics in knee arthroscopy has been established as the standard procedure for many years. As early as the 1990s, needle arthroscopy was occasionally used as a diagnostic tool. In addition to the development of conventional optics technology in terms of camera and resolution, needle arthroscopes are now available with chip-on-tip image sensor technology. To date, no study has compared the performance of this kind of needle arthroscopy versus standard arthroscopy in the clinical setting in terms of the visibility of anatomical landmarks.

In this monocentric prospective feasibility study, our aim was to evaluate predefined anatomical landmarks of the knee joint using needle arthroscopy (0° optics) and conventional knee arthroscopy (30° optics) and compare their performance during knee surgery.

**Methods:**

Examinations were performed on eight cadavers and seven patients who required elective knee arthroscopy. Two surgeons independently performed the examinations on these 15 knee joints, so that we were able to compare a total of 30 examinations. The focus was on the anatomical landmarks that could be visualized during a conventional diagnostic knee arthroscopy procedure. The quality of visibility was evaluated using a questionnaire.

**Results:**

In summary, the average visibility for all the anatomic landmarks was rated 4.98/ 5 for the arthroscopy using 30° optics. For needle arthroscopy, an average score of 4.89/ 5 was obtained. Comparatively, the needle arthroscope showed slightly limited visibility of the retropatellar gliding surface in eight (4.5/ 5 vs. 5/ 5), medial rim of the patella in four (4.85/ 5 vs. 5/ 5), and suprapatellar recess in four (4.83/ 5 vs. 5/ 5) cases. Needle arthroscopy was slightly better at visualizing the posterior horn of the medial meniscus in four knee joints (4.9/ 5 vs. 4.85/ 5).

**Conclusion:**

Needle arthroscopy is a promising technology with advantages in terms of minimally invasive access and good visibility of anatomical landmarks. However, it also highlights some limitations, particularly in cases with challenging anatomy or the need for a wide field of view.

## Background

Knee arthroscopy has developed rapidly over the past few decades and is now an established and frequently used surgical procedure for a variety of indications, such as partial medial meniscectomy, chondroplasty, anterior cruciate ligament reconstruction.

This is demonstrated not only by the numbers of the most common surgical procedures in Germany but also by the data from the German Arthroscopy Registry (DART) [1–3]. At the beginning of each arthroscopy procedure, a standardized diagnostic round is initially performed to accurately evaluate the internal structures in order to visualize potential injuries [2, 4].

In terms of the optics used, a large number of different endoscopes are currently available based on the variety of visualization options. However, the use of the 30° optics in knee arthroscopy has been established as the standard procedure for many years. As early as the 1990s, needle arthroscopy/ mini-arthroscopy was occasionally used as a diagnostic tool [5, 6]. At that time, the technology was mostly based on fiberoptic needle arthroscopy. However, comparative studies indicated no better visualization and no superior assessment of intra-articular structures by needle arthroscopy than the standard rigid rod-lens arthroscope [7, 8].

In addition to the development of conventional optics technology in terms of camera and resolution, needle arthroscopes are now available with chip-on-tip image sensor technology, where the optics and sensor are located at the tip. This is one of the most important technical advances in needle arthroscopy, as this technology enables high-resolution visualization despite the small diameter of the needle arthroscope.

Moreover the diagnostic capabilities of needle arthroscopy, its applications in reconstructive techniques of different joints have been demonstrated in recent years [8–14]. Needle arthroscopy may also be used as an alternative in cases where magnetic resonance imaging is contraindicated [15–19]. Needle arthroscopy may also be cost-effective as it requires fewer hospital resources, produces two-thirds less non-recyclable waste and is suitable for in-office treatment [20].

The surgical access used for needle arthroscopy, with an outer diameter of 2.2 mm, is similar to the level of invasiveness of needle puncture/infiltration (18–12G/ 1.2–2.7 mm) of the knee joint, [9]. In contrast, the 30° optics usually have a diameter of 4 mm (5.5–6.5 mm including the arthroscopy shaft).

The currently available literature on the use of needle arthroscopy, in this case the NanoScope™ (Arthrex, Naples, Florida, USA), is still small.[21]. To date, no study has compared the performance of needle arthroscopy versus standard arthroscopy in the clinical setting in terms of the visibility of anatomical landmarks.

This study aimed to evaluate predefined anatomical landmarks of the knee joint using the 30° optics and needle arthroscopes and compare their performance during knee surgery.

## Methods

In this monocentric prospective feasibility study, needle arthroscopy (0° optics) was compared with conventional knee arthroscopy (30° optics). The study protocol was reviewed and approved by the responsible ethics committee (Az. 219/19-ek).

**Fig. 1:**
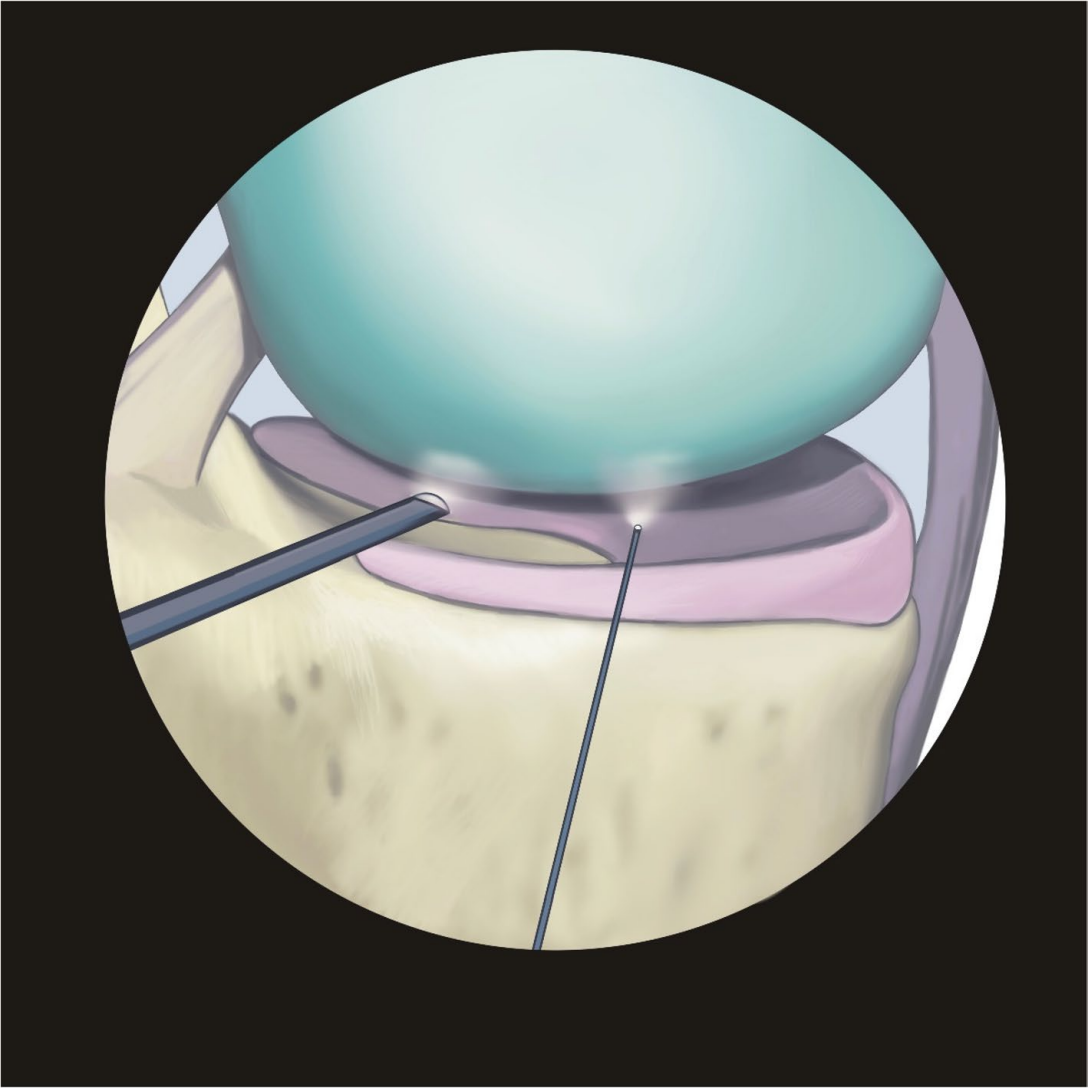
Comparison of size ratios between needle arthroscope and 30° optics (Illustration created by the authors)

We used an arthroscope (NanoScope™, Arthrex (Naples, Florida, USA)) with a needle optic of 0° direction and 120° field of view. The diameter of the needle optics was 1.9 mm (2.2 mm including the arthroscopic shaft). A 30° optic arthroscope (AR-3350–4030, Arthrex (Naples, Florida, USA)) with a diameter of 4.0 mm was used for the comparison (Fig. 1 and Table 1).

**Table 1:** Comparison of the selected technical specifications between needle arthroscope (NanoScopeTM, Arthrex (Naples, Florida, USA)) and the 30° optics

	**NanoScope™**	**30° Optic**
**Diameter of the optic**	1.9 mm	4–4.8 mm
**Diameter including the arthroscopy shaft**	2.2 mm	5.5–6.5 mm
**Direction of view**	0°	30°
**Field of view**	120°	120°

Based on the recommendations of the Association of Statutory Health Insurance Physicians (Kassenärztliche Bundesvereinigung) for the documentation of a knee arthroscopy, twenty predefined anatomical landmarks in different knee joint compartments were used as comparison parameters [22]. The focus was on the anatomical landmarks that could be visualized during a conventional diagnostic knee arthroscopy procedure, and how well could they be evaluated during surgery.

The study was divided into two distinct phases. In the first phase of the test series, two surgeons (one 10th year senior physician and one 4th year resident) performed the arthroscopic procedures at the Institute of Anatomy on cadavers, which were preserved using “Thiel fixation”. In order to examine a larger number of knee joints, we therefore extended this study to include the knee joints of the cadavers for practical reasons, as the needle arthroscope we used is a disposable device. The knee joints of the thiel-fixed cadavers are haptically almost identical to human ones [23, 24].

In the second phase, patients who were enrolled for elective knee arthroscopy during the study period of 4 weeks were included in the study. The surgeons performed the examinations independently.

The inclusion criteria were planned elective knee arthroscopy and the presence of both participating surgeons. The patients were also educated about the study prior to the surgery. The exclusion criteria were preoperatively diagnosed avulsion fractures, knee dislocations, presence of hemarthrosis, inflammatory lesions, and knee joint empyema.

**Fig. 2:**
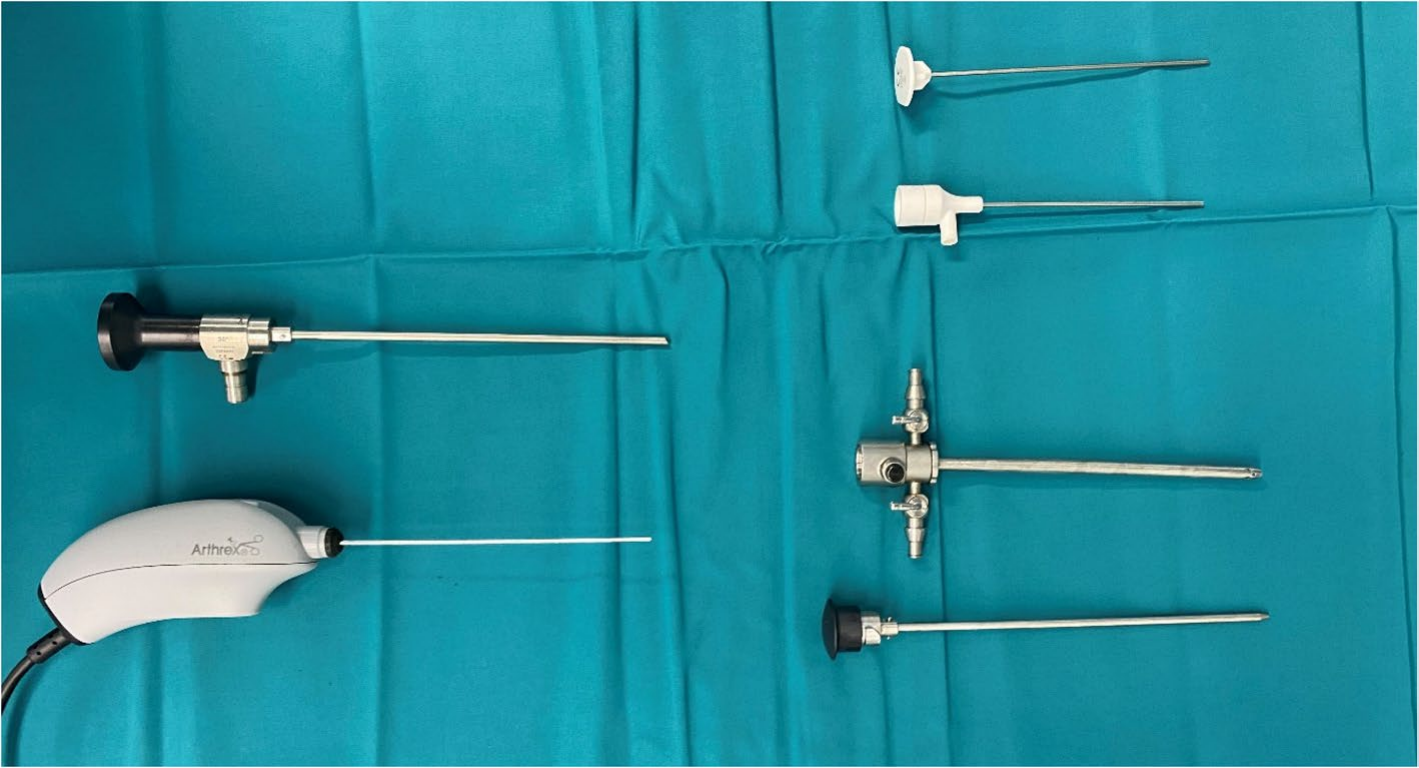
Instrument set for diagnostic arthroscopy with the NanoScope.™ (Arthrex (Naples, Florida, USA)) handpiece and inflow cannula, as well as the 30° optics, arthroscopy shaft, and obturators (Illustration created by the authors)

Preoperative preparations were performed according to the general standards for conventional knee arthroscopy such as checking for indication/contraindication, obtaining informed consent via consent forms, surgical preparations, and team timeout calling**.** The needle arthroscope and related instruments were sterilized and placed on a separate table (Fig. 2). The leg to be treated was positioned at 90° in the leg holder, and the other leg was splayed out. The knee joints of cadavers, in the first phase, were positioned in the same manner. We used the high anterolateral and medial portals for surgical access.

According to the study protocol, arthroscopy was initially performed using the needle arthroscope, followed by the conventional 30° optics one. To enhance comparability of the results and eliminate the potential for bias resulting from anatomical variations in the limited number of participants, we performed both techniques consecutively on one knee each. The diagnostic round in this study was similar to the standardized round at the beginning of diagnostic knee arthroscopy. In addition to detailed photo and video documentation, the quality of visibility of the relevant anatomical landmarks was evaluated using a questionnaire. A Likert scale was used to grade five different categories from "1 = poor" to "5 = very good." The highest score was given if the anatomical structure, as well as possible pathological lesions, could be completely visualized, and evaluated without limitations. In contrast, an impossible visualization of the anatomical landmark was defined as "poor" (Table 2).

**Table 2:** Likert scale for rating the visualization of the corresponding anatomical landmarks

5–Very good	Complete visualization without additional maneuvers
4–Good	Good visualization without additional maneuvers
3–Satisfactory	Good visualization due to additional maneuvers
2–Sufficient	Visualization possible only to a limited extent despite additional maneuvers
1–Poor	Visualization not possible despite additional maneuvers

The prepared study protocol and the associated questionnaire were available in duplicates for each case.

The data were analyzed using descriptive statistics, which included means of standard deviations for continuous variables and numbers as percentages for categorical variables. All statistical analyses were performed using SPSS Statistics 26 (IBM, Armonk, New York, USA).

## Results

Examinations in the first phase of the test series were performed on eight cadavers and in the second phase on seven patients (Mean age 42.43 (SD = 12.83) 4 females, 3 males) who required elective knee arthroscopy. This means that both surgeons independently performed examinations on 15 different knee joints, so that we were able to compare a total of 30 examinations.

In summary, the average visibility for all the anatomic landmarks was rated 4.98/ 5 for the conventional arthroscopy using 30° optics. For needle arthroscopy, an average score of 4.89/ 5 was obtained. Overall, both 30° optics and needle arthroscopy examinations showed impressive visibility of the previously defined anatomical landmarks (Table 3).

**Table 3:** Anatomical landmarks with corresponding visibility by the 30° optic and the needle arthroscope

Medial compartment		Central compartment		Lateral compartment		Patellofemoral compartment	
30° optic	needle arthroscope	30° optic	needle arthroscope	30° optic	needle arthroscope	30° optic	needle arthroscope
Posterior horn of the medial meniscus		Intercondylar region (ACL^a^/ PCL^b^)		Posterior horn of the lateral meniscus		Femoral trochlea	
4.87	4.90	5.0	5.0	5.0	4.73	5.0	4.97
Root of the posterior horn of medial meniscus		Tibial insertion ACL with ligamentum transversum		Root of the posterior horn of lateral meniscus		Patellofemoral joint	
4.85	4.90	5.0	5.0	5.0	4.73	5.0	4.53
Medial meniscus with pars intermedia and anterior horn		Tibial insertion of the PCL		Anterior horn of the lateral meniscus		Medial patellar rim with medial trochlear rim	
5.0	5.0	5.0	4.98	5.0	5.0	5.0	4.85
Medial recess		Femoral insertion of the ACL		Lateral joint space with popliteus tendon		Lateral patellar rim with lateral trochlear rim	
5.0	4.90	5.0	5.0	5.0	4.93	5.0	4.90
Posteromedial compartment / dorsomedial recessus				Popliteus tendon		Patellar apex and central trochlea	
5.0	4.90			5.0	4.86	5.0	4.98
						Suprapatellar recess	
						5.0	4.83

^a^Anterior cruciate ligament

^b^Posterior cruciate ligament

Differences were observed in some selected structures, which could be better visualized using the conventional 30° optics. Comparatively, the needle arthroscope showed slightly limited visibility of the retropatellar gliding surface in eight (4.5/ 5 vs. 5/ 5), medial rim of the patella in four (4.85/ 5 vs. 5/ 5), and suprapatellar recess in four (4.83/ 5 vs. 5/ 5) cases. However, needle arthroscopy was slightly better at visualizing the posterior horn of the medial meniscus in four knee joints (4.9/ 5 vs. 4.85/ 5).

For one knee joint, both surgeons were unable to evaluate the posterior horn of the lateral meniscus due to the presence of clear hypertrophic scar tissue. However, in all the other joints, this structure was clearly visible without restriction. In another case, the medial meniscus was dislocated and therefore, could only be evaluated without restriction after reduction the medial meniscus during conventional arthroscopy. The trocar of the needle arthroscope was marginally deformed in two cases during the cadaver study. In both cases, this was caused by severe scarring or severely constricted anatomy. Additionally, slippage of the trocar due to non-existent fixation also led to short-term limited visibility without any resulting complications. At the beginning of each examination, there was a prolonged time interval until sufficient water pressure was available; therefore, the visibility was temporarily limited at the beginning.

## Discussion

This study aimed to evaluate which anatomical landmarks in the knee joint were visible with different optics (30° vs. needle arthroscope) and how well they were assessed intraoperatively.

With needle arthroscopy, the anatomical landmarks in the knee joint could be well visualized and assessed during the diagnostic round.

This finding is in contrast to comparative studies with fiberoptic systems from the 1990s. These showed inferiority with regard to the assessability of intra-articular structures [7, 25]. Furthermore, the chip-on-tip image sensor technology of needle arthroscopy represents a technological development that includes both technological and ergonomic quality advantages compared to fiberoptic systems. There are studies showing that the evaluation of a knee joint during needle arthroscopy can be equivalent to a conventional arthroscopic examination and more accurate than an magnetic resonance imaging [16, 17]. However, the currently available literature is still limited [21]. In our study certain anatomical structures, especially in the retropatellar region, could only be visualized to a limited extent by needle arthroscopy. This is most likely explained by the general characteristics of the 0° optic and the associated lack of possibility of enlarging the field of view by rotating the optic, which is possible with the 30° optic. Owing to the smaller diameter of the 0° optic and the associated flexibility, less manipulation of the knee joint is required for visualization of the anatomical structures, which also explains the slight advantage in visualizing the posterior horn of the medial meniscus. In this study, the presence of hemarthrosis, inflammatory changes, and knee joint empyema were considered under exclusion criteria because of the poor visibility associated with low flow volume compared to conventional arthroscopy.

Overall, the needle arthroscope is a compact and transportable imaging system and offers good visibility of anatomical landmarks. Additionally is its minimally invasive access comparable to the invasiveness of a needle puncture or therapeutic infiltration of the knee joint that does not require skin sutures. Therefore, the use for diagnostic and limited therapeutic indications in the outpatient setting could be of interest. In contrast, in some mechanical problems occurred in the cadaver study because of the low material thickness due to pronounced scarring or constricted anatomical structures. It is possible that the low material thickness and limited mechanical strength will restrict the range of indications in the future. Ideal indications could therefore be diagnostic arthroscopies and, with limited mechanical stress, also therapeutic procedures. This has already been published for the knee joint in recent years [9, 19, 21, 26, 27]. Also conceivable is the targeted application of biologics (e.g. PRP, hyaluron) under visual control of the injured structure.

For a better overview, we have listed a summary of the results, including advantages and disadvantages of the needle arthroscope optics in a table (Table 4).

**Table 4:** Summary of the results, including advantages and disadvantages of the needle arthroscope

**Advantages**	
Good Visibility	The study shows that needle arthroscopy provides good visibility of anatomical landmarks in the knee joint, comparable to conventional arthroscopy in most cases
Minimally Invasive	Needle arthroscopy offers a minimally invasive approach, which can be beneficial for diagnostic and limited therapeutic indications, reducing the need for skin sutures
Compact and Transportable	Needle arthroscopes are compact and transportable imaging systems, making them potentially suitable for outpatient settings
Technological Advancements	The use of chip-on-tip image sensor technology in needle arthroscopy represents a technological advancement, offering better image quality and ergonomic advantages compared to older fiberoptic systems
**Disadvantages**	
Limited Field of View	Needle arthroscopy with 0° optics has a limited field of view compared to conventional arthroscopy with 30° optics. This limitation can affect the visualization of certain anatomical structures, particularly in the retropatellar region
Mechanical Problems	The study reports instances of mechanical problems with needle arthroscopy, such as trocar deformation and slippage, especially in cases with pronounced scarring or constricted anatomical structures
Material Thickness	The material thickness of the needle arthroscope may limit its use for certain procedures, particularly those involving mechanical stress, such as meniscus resection or cartilage therapy
Small Study Size	The study acknowledges a small number of cases, which may limit the generalizability of the findings
Exclusion Criteria	The exclusion criteria in the study, including the presence of hemarthrosis, inflammatory changes, and knee joint empyema, could affect the applicability of needle arthroscopy in real clinical scenarios

In future studies, it is essential to conduct prospectively recruited observational studies and randomized controlled trials, ensuring the use of comparable outcome measures. Clinical studies should particularly emphasize the utilization of objective and patient-reported outcome measures to evaluate clinical efficacy.

### Limitations

The limitations of the study include the small number of cases. We also pooled data from cadavers and patients, and compared the mean values. In addition, no invasive procedures such as meniscus resection or cartilage therapy were performed with the needle arthroscope in our study.

## Conclusion

Overall, the study suggests that needle arthroscopy is a promising technology with advantages in terms of minimally invasive access and good visibility of anatomical landmarks. However, it also highlights some limitations, particularly in cases with challenging anatomy or the need for a wide field of view. Further research and clinical experience may provide a better understanding of the specific indications and limitations of needle arthroscopy in knee surgery.

## Data Availability

The datasets used and/or analysed during the current study are available from the corresponding author on reasonable request.
